# An expression screen for aged-dependent microRNAs identifies miR-30a as a key regulator of aging features in human epidermis

**DOI:** 10.18632/aging.101326

**Published:** 2017-11-19

**Authors:** Charlotte Muther, Lara Jobeili, Maëlle Garion, Sandrine Heraud, Amélie Thepot, Odile Damour, Jérôme Lamartine

**Affiliations:** ^1^ Laboratory of Tissue Biology and Therapeutic Engineering, CNRS UMR5305, University Claude Bernard Lyon I, F69367 Lyon, France; ^2^ LabSkin Creations, F69003 Lyon, France; ^3^ Banque de Tissus et Cellules, Hospices Civiles de Lyon, F69003 Lyon, France

**Keywords:** microRNA, skin, epidermis, aging, differentiation, apoptosis

## Abstract

The mechanisms affecting epidermal homeostasis during aging remain poorly understood. To identify age-related microRNAs, a class of non-coding RNAs known to play a key role in the regulation of epidermal homeostasis, an exhaustive miRNA expression screen was performed in human keratinocytes from young or elderly subjects. Many microRNAs modulated by aging were identified, including miR-30a, in which both strands were overexpressed in aged cells and epidermal tissue. Stable MiR-30a over-expression strongly impaired epidermal differentiation, inducing severe barrier function defects in an organotypic culture model. A significant increase was also observed in the level of apoptotic cells in epidermis over-expressing miR-30a. Several gene targets of miR-30a were identified in keratinocytes, including *LOX* (encoding lysyl oxidase, a regulator of the proliferation/differentiation balance of keratinocytes), *IDH1* (encoding isocitrate dehydrogenase, an enzyme of cellular metabolism) and *AVEN* (encoding a caspase inhibitor). Direct regulation of *LOX*, *IDH1* and *AVEN* by miR-30a was confirmed in human keratinocytes. They were, moreover, observed to be repressed in aged skin, suggesting a possible link between miR-30a induction and skin-aging phenotype. This study revealed a new miRNA actor and deciphered new molecular mechanisms to explain certain alterations observed in epidermis during aging and especially those concerning keratinocyte differentiation and apoptosis.

## INTRODUCTION

Whatever their structure and anatomic location, tissues are vulnerable to injury and, inevitably, to aging. Skin is a prominent model for studying organ and tissue aging. Skin aging can be modulated by both intrinsic genetically programmed factors and extrinsic environ-mental factors such as pollution, climatic conditions or sun exposure. Intrinsic, or “chronological”, aging is the major cause of skin thinning, impacting both epidermal and dermal compartments. Dermal thinning is mainly caused by disaggregation of collagen and elastin fibers, whereas epidermal atrophy is the result of progressive slowing of proliferation and age-related differentiation defects. UV radiation during sun exposure is the main actor in extrinsic skin aging, by direct action on nucleic acids or through generation of strong oxidative stress, damaging DNA, proteins and lipids. Moreover, UV radiation activates signal transduction pathways, leading to immune response activation, cellular senescence and tissue degradation [1]. The combination of intrinsic and extrinsic factors leads to progressive functional defects in aged skin, including impaired barrier function, delayed wound healing, and impaired cutaneous im-mune response and sensory function. Schematically, age-related skin changes can be considered as a progressive loss of tissue homeostasis, especially in the epidermis, where the balance between keratinocyte proliferation and differentiation is carefully controlled. In the epidermis, keratinocytes proliferate in the basal layer, which is the regenerative layer, and then differentiate progressively in suprabasal layers up to a terminally differentiated state in the horny layer, where the keratinocytes known as corneocytes generate a rigid structure ensuring the barrier function of the skin. We and others have previously identified key regulators of this balance, and especially transcription factors such as GATA3, RUNX1 and P63 [2–4], some of which are dis-regulated in aged skin [5]. In the past ten years, non-coding RNAs emerged as additional regulators of epidermal homeostasis, with the identification of numerous long non-coding RNAs [6] and microRNAs [7, 8] involved in the control of epidermal differentiation. MicroRNAs (miRNAs) are short non-coding RNAs involved in almost all cellular processes, including proliferation, differentiation, cell-cell communication and stress response. They are single strand RNAs, between 21 and 24 nucleotides in length, able to regulate gene expression post-transcriptionally through direct binding to their target messenger RNA, usually to the 3′ un-translated region of the mRNA. The involvement of miRNAs in animal aging was first demonstrated in mice, where a conditional knock-out of Dicer, a key protein in miRNA biogenesis, led to rapid onset of aging and cellular senescence [9]. Moreover, senescent associated microRNAs, identified in keratinocytes during replicative or stress-induced senescence [10], may play a role in skin aging. However, the complex aging process is not restricted to cellular senescence, and the involvement of microRNAs in chronological epidermal aging remains poorly understood. To go further into this question, we set up a large-scale transcriptional analysis to identify miRNAs that are up- or down-regulated in keratinocytes during aging and could be involved in the emergence of the epidermal aging phenotype. We identified numerous microRNAs with age-dependent expression in keratino-cytes. The study focused on miR-30a, and investigated its function in keratinocytes by stable over-expression and generation of reconstructed epidermis. We also identified new targets of miR-30a, three of which were potentially involved in the functional defects observed in aged epidermis.

## RESULTS

### Identification of aged-related microRNAs in human keratinocytes

To identify miRNAs exhibiting age-modulated expression, a genome-wide expression analysis was performed of primary human keratinocytes cultured from skin biopsies of healthy infants (3-6 years old, n=4), young adults (20-40 years old, n=4) and aged adults (60-71 years old, n=4). Cultured keratinocytes are a valuable model for expression screening in epidermal function, as they retain some features of the tissue they are extracted from, especially in aging and the senescence phenotype [11].

**Figure 1:**
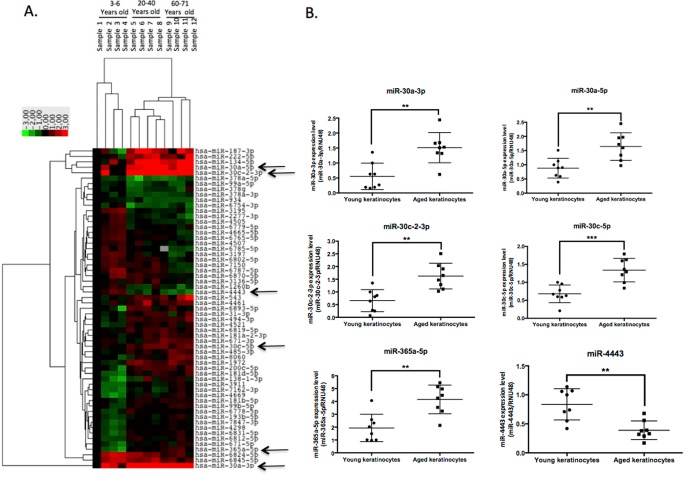
A large-scale expression screen identifies microRNAs modulated by aging in human keratinocytes (**A**) Hierarchical clustering of miRNA differentially expressed in keratinocytes from four infants (3-6 years), four young adult (20-40 years) or four aged adults (60-71 years). Green color corresponds to underexpression and red color to overexpression. The data are normalized to the first infant sample. (**B**) QPCR Validation of 6 modulated microRNAs indicated by arrows in the clustering data: miR-30a-3p, miR-30a-5p, miR-30c-2-3p, miR-30c-5p, miR-365a-5p and miR-4443 expression levels were analyzed by QPCR in 8 independent cultures of keratinocytes from young and aged donors. Results are mean +/− SD from three independent samples. **P<0,01, ***P<0,001.

A miRNA microarray screening identified 60 miRNAs significantly modulated (p<0.05, fold change > 1.5) between at least 2 of the 3 sample groups analyzed (Table 1 and Figure 1A). Some of these age-associated miRNAs have been previously shown to be induced in senescent or aged keratinocytes such as miR-138, miR-181a and miR-181b [10], strengthening the relevance of our model. Most of the modulated microRNAs were expressed differentially between the youngest group (3-6 years) and the 2 adult groups, suggesting a specific microRNA pattern in keratinocytes prepared from child skin. Six modulated microRNAs were then selected for real-time PCR validation, which confirmed significant induction of miR-30a-3p and -5p, miR-30c-5p and miR-30c2-3p and miR-365a-5p in keratinocytes from aged skin, whereas miR-4443 was significantly reduced in the aged sample (Figure 1B).

**Table 1:** Human microRNAs significantly modulated (p<0.05 FC > 1.5) between at least 2 of the 3 groups of samples analyzed

	Aged vs Child		Adult vs Child		Aged vs Adult	
microRNA	FC	p-value	FC	p-value	FC	p-value
hsa-miR-1260b	−1,87	0,0125				
hsa-miR-134-5p			4,69	0,025		
hsa-miR-138-1-3p	2,22	0,027				
hsa-miR-181a-2-3p	1,61	0,0199				
hsa-miR-181b-5p	1,54	0,0199				
hsa-miR-181d-5p	2,40	0,021	2,18	0,022		
hsa-miR-187-3p			7,27	0,016		
hsa-miR-193b-5p	1,62	0,037				
hsa-miR-1972	2,37	0,015	2,74	0,0033		
hsa-miR-200c-5p	2,20	0,0154	2,07	0,013		
hsa-miR-222-5p			4,77	0,0058		
hsa-miR-2277-3p	−2,15	0,0256				
hsa-miR-30a-3p	6,78	0,009	5,96	0,00411		
hsa-miR-30a-5p	4,65	0,007	4,4	0,0041		
hsa-miR-30c-2-3p	5,19	0,023	5,44	0,00547		
hsa-miR-30c-5p	1,92	0,01	1,78	0,0127		
hsa-miR-31-3p			1,6	0,0237		
hsa-miR-3136-5p					−1,56	0,034
hsa-miR-3195	−2,43	0,027				
hsa-miR-3197	−2,26	0,017				
hsa-miR-365a-5p	2,65	0,0074	1,88	0,021		
hsa-miR-378a-3p			−1,56	0,0111		
hsa-miR-378a-5p			−2,07	0,0177		
hsa-miR-378g			−1,53	0,0247		
hsa-miR-3911	1,92	0,04				
hsa-miR-4298	1,59	0,02088	1,54	0,0196		
hsa-miR-4443	−3,65	0,004	−2,27	0,0187		
hsa-miR-4461	1,88	0,0173				
hsa-miR-4505	−2,05	0,012				
hsa-miR-4507	−1,59	0,0157				
hsa-miR-4521			1,69	0,0119		
hsa-miR-4665-5p	−1,75	0,027				
hsa-miR-4669	2,35	0,017				
hsa-miR-485-3p	1,91	0,0161				
hsa-miR-494-3p			1,84	0,00194		
hsa-miR-543			1,89	0,006		
hsa-miR-671-3p	1,95	0,0036			1,69	0,0198
hsa-miR-671-5p	1,81	0,0389				
hsa-miR-6754-3p			−2,13	0,016		
hsa-miR-6765-5p	−1,71	0,0346				
hsa-miR-6778-5p			1,63	0,0407		
hsa-miR-6779-5p			−1,5	0,0362		
hsa-miR-6785-5p	−2,04	0,026			−1,56	0,0127
hsa-miR-6787-5p					−1,72	0,027
hsa-miR-6802-5p	−1,77	0,028			−1,56	0,01149
hsa-miR-6802-5p					−1,51	0,0114
hsa-miR-6812-5p	1,68	0,0355	1,59	0,044		
hsa-miR-6819-5p			1,5	0,0162		
hsa-miR-6824-5p					−2	0,012
hsa-miR-6831-5p	1,83	0,014	1,69	0,019		
hsa-miR-6845-5p					−1,78	0,0352
hsa-miR-6870-5p					−1,51	0,0123
hsa-miR-6893-5p			2,19	0,0377		
hsa-miR-7150	−1,84	0,029				
hsa-miR-7162-3p	1,93	0,049				
hsa-miR-7847-3p	1,64	0,03				
hsa-miR-8060	2,43	0,014			2,06	0,025
hsa-miR-934	−1,73	8,48E-05				
hsa-miR-99a-5p			−1,58	0,025		
hsa-miR-99b-5p	1,58	0,0286				

The fold changes (FC) indicated in this table correspond to the mean FC between the 2 groups of 4 samples. Empty squares correspond to non-significant modulation between the 2 groups of samples. The p-values correspond to multiple t-test with a FDR (False Discovery Rate) of 5%.

Aged: keratinocytes from 60-71 years skin samples; Adult: keratinocytes from 20-40 years skin samples; Child: keratinocytes from 3-6 years skin samples.

### MiR-30a-3p and miR-30a-5p are induced in aged epidermis

**Figure 2:**
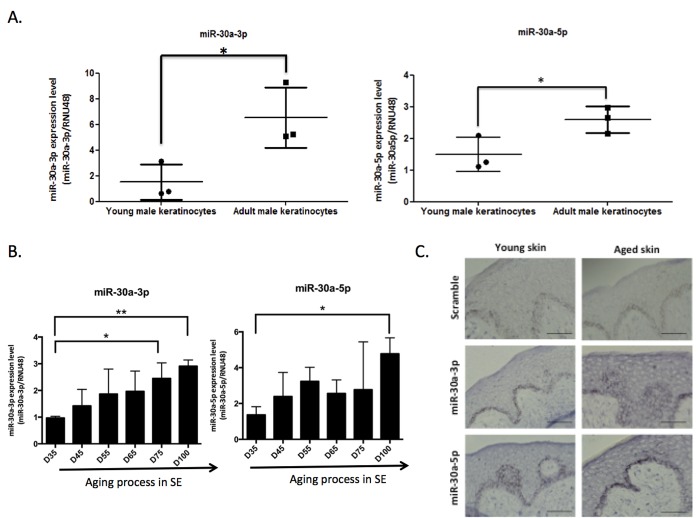
MiR-30a expression level during aging The expression levels of miR-30a-3p and miR-30a-5p were evaluated by QPCR in (**A**) keratinocytes from infant and adult male donors and (**B**) skin equivalent (SE) model mimicking aging with a long time of culture: MiR-30a expression was monitored along the aging process from day 35 (D35) to day 100 (D100) of culture. For QPCR: results are mean +/− SD from three independent samples. *P< 0,05, **P<0,01. (**C**) The expression levels and the localization of miR-30a-3p and miR-30a-5p were evaluated by *in situ* hybridization in young and aged skin. Scale bar = 50 μm. Representative pictures were shown.

We then focused on miR-30a, both strands of which are induced in keratinocytes from aged skin. We first checked that the modulation observed in the initial screening was not gender-related: we confirmed the overexpression of miR-30a-3p and miR-30a-5p in 3 samples of keratinocytes from adult male skin (30-50 years old) compared to keratinocytes from child foreskin (Figure 2A). To further confirm the impact of aging on miR-30a expression, independently of parameters such as the anatomic location or gender of the skin biopsy used to prepare the keratinocytes, miR-30a-3p and -5p expression was analyzed in RNA samples from long-term culture of skin equivalents mimicking chronological aging [12]. There was prog-ressive increase in both miR-30a strands during culture, with a significant difference between D35 and D100 for both miR-30a-3p and -5p (Figure 2B). Finally, miR-30a expression in human skin was addressed by *in situ* hybridization of miR-30a-3p and -5p probes: there was clear induction of miR-30a-5p and -3p in aged skin, with staining throughout the epidermis. The expression of miR-30a-3p was faint in young skin (Figure 2C), reflecting the lower expression of the -3p compared to the 5p strand observed in cultured keratinocytes on quantitative PCR (Figure S1). We also confirmed miR-30a-3p induction in aged epidermis by QPCR (Figure S2). Taken together, these results support aged-related induction of miR-30a-3p and miR-30a-5p in cultured keratinocytes and epidermal tissue.

### MiR-30a over-expression impairs epidermal differentiation

**Figure 3:**
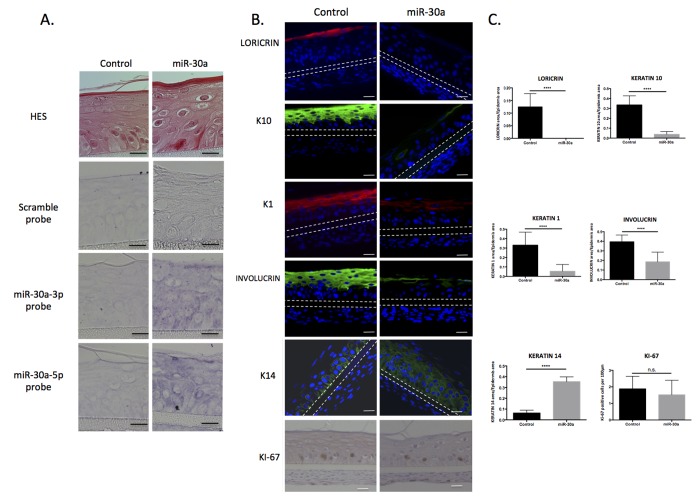
Caracterization of reconstructed epidermis overexpressing miR-30a Reconstructed epidermis (REs) were generated from keratinocytes transduced with the miR-30a lentiviral construction and treated by doxycycline to activate miR-30a expression. (**A**) Evaluation of the morphology of the REs by HES staining and confirmation of the miR-30a overexpression by *in situ* hybridization in control and miR-30a RE. (**B**) Immunofluorescent staining of differentiation markers (LORICRIN, K10, K1, INVOLUCRIN and K14) and immunohistochemical staining of proliferation marker (Ki67) in control and miR-30a overexpressing REs. Counterstaining was performed with DAPI and the polycarbonate membrane is indicated by dotted lines. (**C**) Quantification of the LORICRIN, K10, K1, INVOLUCRIN and K14 labeled area in the REs and evaluation of the number of KI67-positive cells. Results are mean +/− SD from three independent samples. ****P<0,0001. ns : non-significant. Scale bar = 25 μm. Representative photographs of 3 independent replicates were shown.

We then investigated the function of miR-30a in keratinocytes. The human miR-30a gene was cloned into a lentiviral based-vector, allowing stable over-expression of both miR-30a-3p and 5p after infection of primary human keratinocytes and stimulation by doxy-cycline. Transduced keratinocytes were then used to generate 3D-reconstructed epidermis, which is a more realistic model of differentiation than monolayer culture. After 3 days' immersed culture, transduced keratinocytes with the miR-30a or the control vector were placed at the air/liquid interface for 14 days and transgene expression was continuously stimulated by doxycycline treatment. *In situ* hybridization confirmed strong induction of miR-30a-3p and -5p in the whole epidermal tissue (Figure 3A). The level of miR-30a-3p and -5p expression was also monitored by QPCR, and showed a mean 10-15-fold increased induction for both strands (Figure S3). Although epidermis thickness was not impacted (data not shown), examination of HES-stained tissue sections revealed an abnormal epidermis in miR-30a-overexpressing tissue, with parakeratosis, big round cells in suprabasal layers and absence of granular layer (Figure 3A). Immunofluorescence stain-ing of several early (K1, K10) and late (INVOLUCRIN, LORICRIN) differentiation markers confirmed that the differentiation process was severely impaired, with absence of staining for the granular marker LORICRIN and strongly reduced expression of K1, K10 and INVOLUCRIN. On the contrary, basal keratin K14 was expressed in almost all the miR-30a-over-expressing epidermis (Figure 3B and 3C) indicating that the differentiation process was repressed and that immature nucleated keratinocytes were still present in the suprabasal layers. Finally, keratinocyte proliferation was assessed by Ki67 immunohistochemical analysis: proliferative cells were restricted to the basal layers in both control and miR-30a-overexpressing epidermis, with no significant difference (Figure 3B and 3C). The results clearly showed that the epidermal differentiation program was impaired in miR-30a-overexpressing tissue, whereas the proliferation process was not modified.

**Identification of miR-30a gene targets in human epidermis**

**Figure 4:**
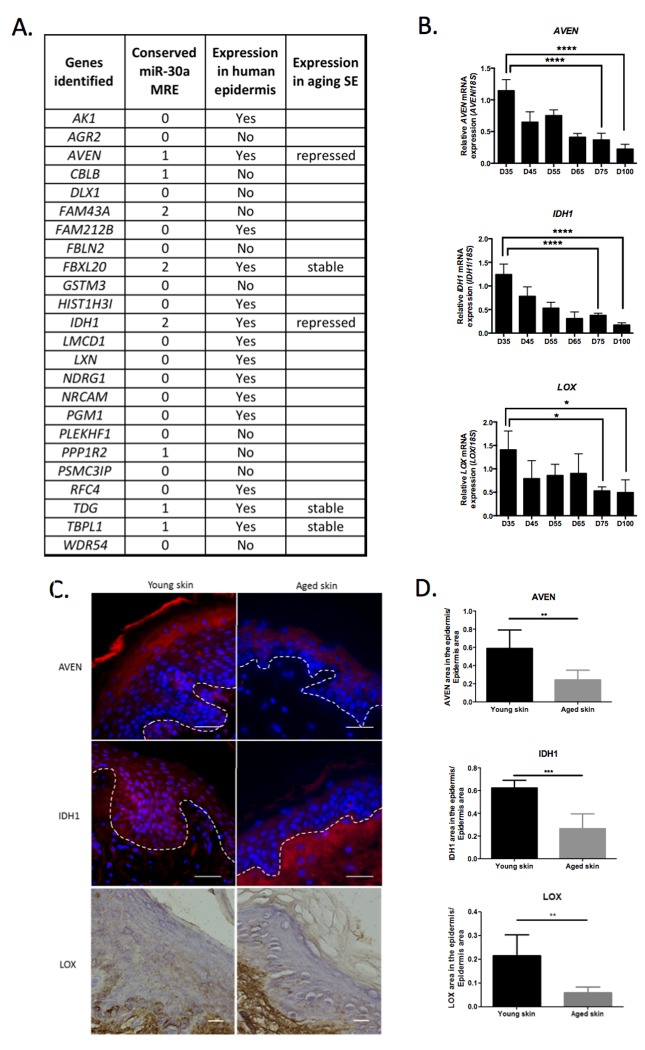
Identification of potential miR-30a targets in human epidermis (**A**) A list of genes potentially targeted by miR-30a according to transcriptome data available in the GEO database. The number of conserved miR-30a MRE is indicated for each gene. The potential expression of these genes in human epidermis (according to the Human Protein Atlas database) is indicated. Five genes exhibiting a least one miR-30a conserved MRE and expressed in human epidermis, were analyzed by QPCR in RNA samples from skin equivalent (SE) model mimicking aging. The expression profile is indicated for these 5 genes. (**B**) The expression levels of *AVEN*, *IDH1* and *LOX* were evaluated by QPCR in RNA samples from SEs mimicking aging with a long time of culture from day 35 (D35) to day 100 (D100). Results are mean +/− SD from three independent samples. *P< 0,05, ****P<0,0001. (**C**) Expression of AVEN, IDH1 or LOX in young or old human skin sections assessed by immunofluorescence for AVEN and IDH1 and by immunohistochemistry for LOX. (D) Quantification of the AVEN, IDH1 and LOX labeled area in the epidermis from young or old human skin sections. Results are mean +/− SD from three independent samples. ***P<0,001, **P<0,01.

To decipher the molecular networks controlled by miR-30a in epidermis, we investigated potential gene targets. Transcriptome data already deposited in the Gene Expression Omnibus (GEO) database on NCBI iden-tified 5 transcriptome projects in which miR-30a expression was modulated (overexpression or silencing) in cultured cells. The potential targets (transcripts repressed after miR-30a overexpression or induced after miR-30a silencing), identified in at least 2 of the 5 transcriptomes, were selected for further analysis. Twenty-four genes were thus identified, 14 of which were potentially expressed in human epidermis accord-ing to the Human Protein Atlas (HPA) database and 8 of which exhibited conserved miR-30a response element (MRE) in their 3′-UTR (Figure 4A). We therefore selected 5 genes (*AVEN*, *FBXL20*, *IDH1*, *TDG*, *TBPL1*) with at least 1 conserved miR-30a MRE and potentially expressed in the epidermis according to the HPA database. Each of these was then tested on RNA samples from long-term culture of skin equivalents mimicking chronological aging. There was a strong decrease in *AVEN* and *IDH1* transcript expression in the late phase of culture, suggesting that these 2 genes might be repressed in aged epidermis (Figure 4B). We also observed that IDH1, AVEN and LOX were re-pressed at the mRNA and protein level in keratinocytes from aged skin (Figure S4). We then selected *AVEN*, encoding a caspase inhibitor, and *IDH1* encoding the isocitrate dehydrogenase 1, as putative miR-30a targets potentially involved in human epidermis aging. The study also focused on *LOX*, encoding a lysyl oxidase known to be involved in keratinocyte differentiation [13] and to be directly regulated by miR-30a in cancer cells [14]. Expression analysis of the *LOX* transcript in the skin equivalent model of aging showed significant decrease in aged tissues (Figure 4B). Finally, we analyzed the expression of the 3 miR-30a targets LOX, IDH1 and AVEN in aged human skin and observed a global reduction in the levels of these proteins in aged epidermis, especially for IDH1 and AVEN (Figure 4C and 4D). For IDH1, a stronger expression was observed in the dermis of aged skin (Figure 4C) suggesting a possible redistribution of the protein in the aged skin.

**Figure 5:**
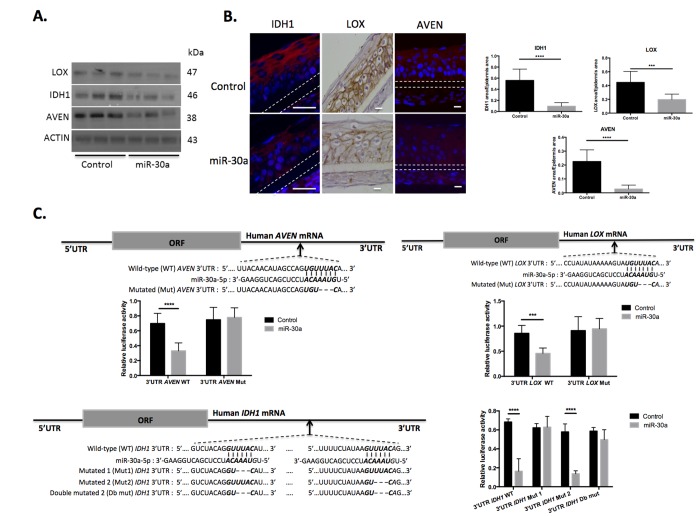
AVEN, IDH1 and LOX are direct targets of miR-30a in keratinocytes (**A**) The expression levels of AVEN, IDH1 and LOX proteins were evaluated by western blotting in cultured keratinocytes transduced by the miR-30a lentivirus construction after doxycycline treatment. (**B**) Immuno-fluorescent staining of AVEN and IDH1 and immunohistochemical staining of LOX in reconstructed epidermis overexpressing or not miR-30a after doxycycline treatment. Counterstaining was performed with DAPI and polycarbonate membrane position is indicated by a dotted line. Representative photographs of 3 independent replicates were shown. Scale bar = 25 μm. Right panels: quantification of the IDH1, LOX or AVEN labeled area in the REs Results are mean +/− SD from three independent samples. ****P<0,0001, ***P<0,001 (**C**) The alignment of miR-30a putative binding sites in human *AVEN*, *IDH1* or *LOX* 3′-UTR region have been schematized to show complementary pairing of miR-30a with *AVEN*, *IDH1* or *LOX* wild-type (WT) and mutant (Mut) 3′-UTR constructs. Transduced keratinocytes were transfected with WT or Mut reporter constructs. Luciferase intensities were normalized to β-galactosidase level. Results are mean +/− SD from three independent samples. *P<0,05, **P<0,01, ***P<0,001, ****P<0,0001.

The study then focused on *IDH1*, *LOX* and *AVEN*, 3 genes potentially targeted by miR-30a and repressed in aged epidermis. To confirm regulation by miR-30a, protein expression in miR-30a-over-expressing kera-tinocytes (Figure 5A) and in reconstructed epidermises with overexpression of miR-30a was studied (Figure 5B): expression of all 3 proteins was strongly reduced in cultured keratinocytes and reconstructed epidermis. To demonstrate direct regulation of *LOX*, *AVEN* and *IDH1* by miR-30a, their 3′ UTR sequence, containing at least 1 specific miR-30a MRE, was cloned into a lucife-rase reporter plasmid. The plasmids were transfected in human keratinocytes previously transduced by the miR-30a lentivirus, and luciferase expression was monitored in the context of miR-30a over-expression. There was a significant reduction in luciferase activity for the 3′-UTR-AVEN WT, 3′ UTR-LOX WT and 3′-UTR-IDH1 WT after over-expression of miR-30a (Figure 5C). Reporter plasmids harboring a modified form of the 3′ UTR for these 3 genes were also constructed, with mutations of the conserved miR-30a MRE (see Figure 5C for the description of WT and mutated 3′ UTR sequences). With mutated forms of the 3′-UTR-AVEN and 3′ UTR-LOX, the effect of miR-30a over-expression on luciferase expression was completely abolished (Figure 5C, top panels). For the 3′-UTR of *IDH1*, the two conserved MREs mutated separately or simultaneously: there was loss of the miR-30a effect on luciferase activity only when the first MRE was mutated (Figure 5C, bottom panels). These data confirmed the specificity of miR-30a's action on these 3 targets by direct binding on specific DNA sequence elements. Finally, we de-monstrated that miR-30a directly targets *LOX*, *IDH1* and *AVEN* genes by specific binding on their 3′UTR.

### Mir-30a regulates barrier function and keratinocytes apoptosis in human epidermis

**Figure 6:**
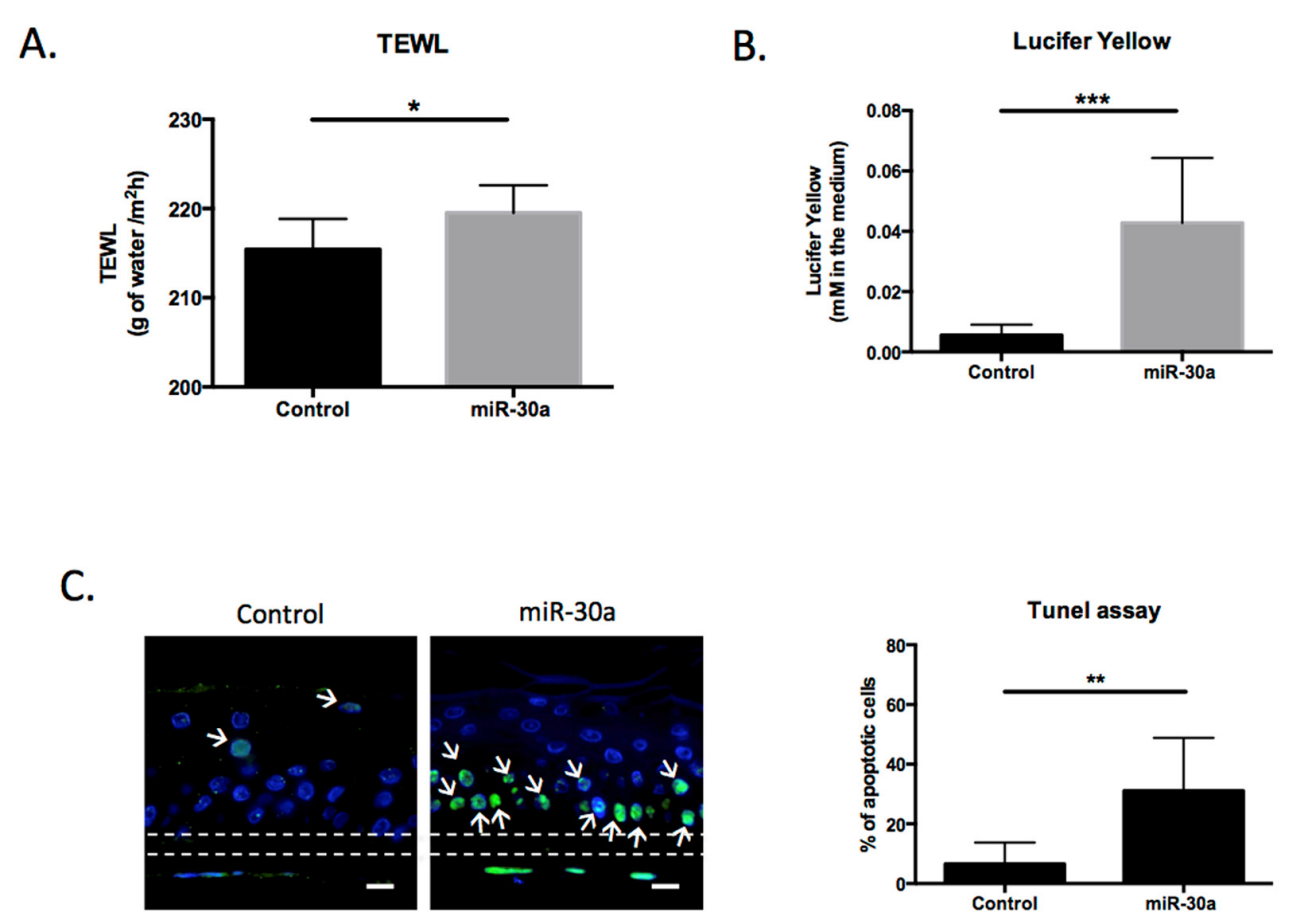
MiR-30a overexpression decreases the functionality of reconstructed epidermis (REs) (**A**) Trans-epidermal water loss (TEWL) was measured for control or miR-30a overexpressing REs after doxycyclin treatment (**B**) Outside-in permeability was evaluated using the Lucifer Yellow assay for control or miR-30a overexpressing REs after doxycyclin treatment (**C**) Apoptosis TUNEL assay for control or miR-30a overexpressing REs after doxycyclin treatment. The apoptotic cells are labeled in green and indicated with white arrows. Scale bar = 25 μm. The Quantification of the % of apoptotic cells appears on the graph. Results are mean +/− SD from three independent samples. *P<0,05, **P<0,01, ***P<0,001.

We demonstrated that miR-30a over-expression impaired expression of keratinocyte differentiation markers in our model of reconstructed epidermis (RE) (Figure 3). Based on these data, we explored the functional consequences of this over-expression on the barrier function. Two complementary tests were performed in control and miR-30a epidermis: trans-epidermal water loss (TEWL) measurement to assess inside-out permeability and a Lucifer yellow assay to evaluate the outside-in permeability of the barrier. There were significant increases in TEWL (Figure 6A) and Lucifer yellow penetration (Figure 6B) in miR-30a-overexpressing tissue, suggesting impaired barrier function. We also demonstrated that miR-30a targeted *AVEN*, a gene encoding an apoptosis inhibitor. We therefore explored apoptosis level in control and miR-30a-over-expressing epidermis: apoptotic cell abun-dance was strongly increased in miR-30a-positive tissue (Figure 6C). Overall, these results indicated that miR-30a overexpression impairs epidermal barrier functionality and increases keratinocyte apoptosis, two defects observed in human aged skin.

## DISCUSSION

In this study, we performed an expression screen in cultured human keratinocytes from young or aged skin to identify age-dependent microRNAs. Since keratino-cytes were extracted from skin with various anatomical locations, we cannot exclude that anatomical differen-ces might impact microRNA expression. However, we confirmed miR-30a induction with aging in a well-recognized *in vitro* model of reconstructed skin mimicking chronological aging [12] where the parameter of anatomic localization is completely abolished. We therefore consider that the main parameter responsible for miR-30a induction in epidermis is the chronological age of the tissue.

We show here for the first time that miR-30a is an aged-dependent microRNA able to impair differentiation and induce apoptosis in human keratinocytes. MiR-30a has been intensively studied in cancer, where it plays a complex and dual role as oncogene or onco-suppressor depending on the type of cancer (for a review: [15].) MiR-30a also participates in a wide range of biological processes in normal cells, including osteogenic and chondrogenic differentiation [16, 17], neuronal develop-ment [18], epithelial-to-mesenchymal transition [19], autophagy [20] and auto-immune response regulation [21]. However, the role of miR-30a in the epidermis remains largely unexplored. Only one paper published to date presented data about miR-30a in keratinocytes: the authors suggested that miR-30a could be regulated by transcription factor P63 and be involved in the onset of differentiation in monolayer cultures of human keratinocytes [22]. Unfortunately, the present culture model was unable to confirm a link between miR-30a and P63 (data not shown), although this could be an important mechanism in the regulation of epidermal homeostasis. The present study is also the first to report miR-30a induction by chronological aging, although a few papers have shown a link between miR-30a expression and cellular senescence, especially in fibroblasts [23] and cord-blood stem-cells [24]. We also observed miR-30a induction in replicative senescent keratinocytes (Figure S5); in our hands, however, over-expression of miR-30a in keratinocytes did not induce senescence, suggesting that miR-30a induction is a consequence but not a cause of cellular senescence in keratinocytes (data not shown).

We demonstrated that 1) miR-30a-5p and -3p are induced in aged epidermis, 2) miR-30a directly targets the *LOX*, *IDH1* and *AVEN* genes, and 3) MiR-30a is able to impair epidermal differentiation and to activate apoptosis when over-expressed in keratinocytes grown in 3-dimensional organotypic culture. It is therefore very tempting to explain miR-30a effects in keratino-cytes in terms of repression of its targets, especially *LOX* and *AVEN*. The *LOX* gene encodes lysyl oxidase, an extracellular enzyme involved in the maturation of connective tissue [25]. It has been also shown that LOX is expressed in human keratinocytes and is able to regulate keratinocyte differentiation independently of its main enzymatic activity [13]. More precisely, silencing LOX by RNA interference strongly impaired terminal differentiation in a reconstructed-epidermis model [13], mimicking the effect of miR-30a overexpression observed in the present study. It can therefore be hypothesized that the negative effect of miR-30a on epidermal differentiation could, at least partly, be linked to LOX repression.

The *AVEN* gene encodes a caspase inhibitor that plays an anti-apoptotic role by interacting with Bcl-XL and Apaf-1 [26]. The role of AVEN in epidermis has been poorly studied. We demonstrated here that AVEN is expressed in human epidermis, is repressed in aged skin and is also down-regulated in RE over-expressing miR-30a and exhibiting a high concentration of apoptotic cells. Taken together, these results suggest that miR-30a-mediated silencing of anti-apoptotic protein AVEN is involved in the apoptosis burst observed in our 3D-cultured keratinocytes. Contrary to LOX and AVEN, the role of IDH1 in the phenotype is not so obvious. *IDH1* encodes isocitrate dehydrogenase, a cytosolic enzyme that catalyzes oxidative decarboxylation of isocitrate to α-ketoglutarate, an important source of NADPH. IDH1 thus plays a key role in anti-oxidative cell protection by maintaining the oxido-reduction potential [27]. IDH1 down-regulation during the early stages of skin tumorigenesis is strongly correlated with tumor promotion [28]; moreover, IDH1 mutations are frequently found in melanoma and various other cancers, including leukemia and glioma, and contribute to metastasis by altering cellular metabolism [29–31]. It has been shown that overexpression of a mutated form of IDH1 in neural stem-cells leads to differentiation defects and increased apoptosis susceptibility [32]. It is therefore possible that down-regulation of IDH1 by miR-30a induces a similar effect in cultured keratino-cytes. In the present study, IDH1 was strongly repressed in aged human epidermis (Figure 4) but also in cultured human keratinocytes from aged individuals (data not shown). IDH1 repression in aged epidermis could therefore contribute to several physiological defects observed in aging tissue, including differentiation defects and increased sensitivity to oxidative stress. Altered IDH1 expression in aged tissues and indivi-duals, already observed in *C. Elegans* [33]*,* may therefore be a general hallmark of aging. However, IDH1 induction in the dermis of old skin (Figure 4C) might also evocate multiple and perhaps opposite functions in the skin during aging.

The present study used 3D organotypic primary keratinocyte culture to mimic human epidermis and to study the functional consequences of miR-30a over-expression. There was a strong increase in apoptotic cell rates in the various layers of the REs and an impairment of epidermal differentiation, correlated with less efficient barrier function. These features, obtained in a simple model of 3D-culture, resemble some aspects of *in situ* epidermis in the elderly. Impairment of epidermal barrier function in aged skin is a recognized functional defect that has been quantitatively documented by several authors, demonstrating that some skin barrier function parameters, such as lipid production and skin surface pH, correlate with age [34–36]. Moreover, it has been shown that the differentiation program is disturbed in aged skin, with reduced expression of various differentiation markers, including loricrin and involucrin [12, 37], two proteins strongly reduced in miR-30a-overexpressing epidermis equi-valents. Finally, aged skin shows increased TUNEL index, with positive cells detected not only in the differentiated terminal layers but also below the granular layer, indicating apoptosis rather than terminal differentiation [38]. The phenotype of the epidermis equivalents obtained with miR-30a-overexpressing keratinocytes can therefore be taken to exhibit some important features of skin aging, and it may be suggested that miR-30a induction in epidermis during aging may be implicated in certain deleterious aspects of aged tissue. MiR-30a thus appears to be a possible target for skin anti-aging strategies using genetic or cosmetic approaches.

## METHODS

### Human skin samples

Normal human skin tissue explants were obtained from the surgical discard of anonymous healthy patients with informed consent of adult donors or children's parents in accordance with ethical guidelines (French Bioethics law of 2004) and declared to the French research ministry (Declaration no. DC-2008-162 delivered to the Cell and Tissue Bank of Hospices Civils de Lyon). Young (<5 years), adult (19-40 years) and aged (>60 years) human primary keratinocytes (HPK) were isolated with trypsin (Gibco, Life Technologies, Carlsbad, CA, USA) and dispase (Dispase II; Roche Diagnostics, Mannheim, Germany), respectively from child foreskin and abdominal or mammary skin biopsies obtained from plastic surgery. Human primary keratino-cytes, isolated as described elsewhere [39] were cultured in KGM2 medium (Promocell, Heidelberg, Germany) as previously described [3] and used at early passage (passage 2 or 3) in the subsequent experiments. All kera-tinocytes were passed in the exponential phase of growth.

### RNA isolation and real-time quantitative PCR

Total RNA was isolated using a mirVana^TM^ miRNA Isolation Kit (Invitrogen, Thermo Fisher Scientific, Vilnius, Lithuania) according to the manufacturer's instructions.

To study miRNA expression profiles, miRNA was reverse-transcribed into cDNA using a TaqMan® MicroRNA Reverse Transcription Kit (Applied Biosystems, Foster City, USA) and analyzed on real-time qPCR using a TaqMan® Universal PCR Master Mix (Applied Biosystems, Foster City, USA) on an AriaMx Realtime PCR system (Agilent Genomics, Santa Clara, CA, USA). Results were normalized to RNU48 snoRNA expression level.

To study mRNA expression profiles, mRNA was reverse-transcribed into cDNA using a PrimeScript^TM^ RT reagent kit (Takara, Shiga, Japan) and analyzed on real-time qPCR using a SYBR® Premix ExTaqII (Takara, Shiga, Japan) on the same instrument. Results were normalized to 18S rRNA expression level. Results were obtained from independent experiments using keratinocytes from 3 different donors. Relative quantification was calculated using the 2ΔΔCt quantification method. The primers are listed in the Supplementary Materials and Methods file.

### Microarray analysis

MicroRNA expression profiles keratinocytes from young (<5 years), adult (19-40 years) and aged skin (>60 years) were analyzed using a whole human genome microarray containing 30,434 probes (GeneChip^TM^ miRNA 4.0 Array; Affymetrix, Santa Clara, CA, USA). RNA amplification, microarray hybridization and scanning, data normalization and analysis were performed at the ProfileXpert genome facility (Lyon, France) as previously described [40]. Significantly modulated microRNAs were selected by fold change ≥ 1.5 and pvalue ≤0.05 (multiple unpaired t-test. FDR = 5%). The microarray data have been deposited into the Gene Expression Omnibus database at NCBI (https://www.ncbi.nlm.nih.gov/geo/) and are available with GEO accession number GSE101493.

### Protein extraction and western blotting

Protein extraction and immunoblotting on nitrocellulose membrane were performed as previously described [41]. The membrane was incubated overnight at 4°C in TBS-T with primary antibody specific to IDH1 (Cell Signaling Technology, Danvers, USA #D2H1, 1/1000), LOX (home-made antibody, 1/250), AVEN (Sigma, St-Louis, USA #HPA020863, 1/100) or ACTIN (Millipore, Billerica, USA #MAB 1501 Clone C4, 1/5000). The membrane was then washed 3 times in TBS-T and incubated with secondary antibody for 1 hour at room temperature. The peroxidase-conjugated secondary antibodies were goat anti-mouse IgG and goat anti-rabbit IgG (Thermo Fisher Scientific, Waltham, USA). Proteins were detected using an enhanced chemiluminescence system (Thermo Fisher Scientific, Waltham, MA, USA) and the signal was detected by the Fusion Fx system (Vilber Lourmat, Collégien, France).

### Plasmid construction, lentiviral production and cell infection

The genomic DNA sequence containing the hsa-miR-30a precursor was amplified with the following primers: 5′- CCGAATTCCCTTGAAGTCCGAGGCAGTA (forward) and 5′- CCGAATTCTACAGAATCGTTGCCTGCAC (reverse). This sequence was cloned into pEN_TTmcs (Addgene #25755, Cambridge, MA, USA) between two EcoRI restriction sites. A control plasmid was constructed with an empty pen_TTmcs. Then, miR-30a or control pen_TTmcs was recombined into pSLIK-Venus (Addgene #25734) using Gateway LR Clonase (Invitrogen, Carlsbad, CA, USA) according to the manufacturer's instructions. All cloned products were sequenced before lentiviral production. Lentiviral vector particles were produced by the vector facility at SFR BioSciences Gerland-Lyon Sud (Lyon, France) as previously described [42].

HKPs were infected at a MOI of 10 and treated with 1 μg/ml doxycycline (Sigma, St-Louis, MO, USA) to induce miR-30a overexpression. For RE production, infected HPKs were treated with 0.1 μg/ml doxycycline. We had previously checked that this low concentration of doxycycline was able to activate miR-30a expression but had no deleterious effect on keratinocytes proliferation and stratification in REs and SEs organotypic models.

### Identification of miR-30a targets and luciferase assays

The Gene Expression Omnibus (GEO) database (https://www.ncbi.nlm.nih.gov/geo/) was used to identify putative targets of miR-30a by comparison of 5 transcriptomes performed on cells after miR-30a mo-dulation (Accession numbers: GSE12908, GSE16569, GSE29921, GSE36565, GSE41607). Genes identified by at least 2 of the 5 transcriptomes were selected for further analysis. The Human Protein Atlas database [43] was then used to select predicted target genes encoding proteins expressed in human epidermis. A final search for conserved miR-30a MicroRNA Recognition Elements (MRE) in the 3′UTR of the selected genes was performed using TargetScan 7.1 (www.targetscan.org).

A 3′-UTR sequence of AVEN (194 pb), IDH1 (240 pb) or LOX (306 pb) containing the candidate miR-30a binding sites was amplified by PCR from human genomic DNA. 3′UTR sequences of LOX and AVEN contained 1 candidate miR-30a conserved binding site and the 3′UTR sequence of IDH1 contained 2. The primers used for this amplification are listed in the Supplementary Materials and Methods file.

After TA-cloning to allow net cuts to restricted sites, the PCR product was cloned into the HindIII and SacI restriction sites downstream of the open reading frame of luciferase in the pMIR-REPORT vector (Ambion, Invitrogen, Carlsbad, CA, USA) to generate AVEN-3′UTR, IDH1-3′UTR or LOX-3′UTR reporter.

A 3 bp deletion in the GTTACA miR-30a binding site was created by site-directed mutagenesis using the QuikChange^TM^ Site-Directed Mutagenesis kit (Stratagene; Agilent Technologies, Santa Clara, CA, USA), with the X-3′UTR reporter vectors as templates and the primers listed in the Supplementary Materials and Methods section. This generated the mut-X-3′UTR reporter vector. All cloned products were sequenced before use.

Luciferase assays were performed on young primary keratinocytes infected by pSLIK Venus control or pSLIK Venus miR-30a using the Dual-Luciferase Reporter Assay (Promega, Madison, WI, USA), according to the manufacturer's instructions. Transient transfection of 500ng pMIR report-3′UTR AVEN, IDH1 or LOX or their mutants and 100ng pMIR β-galactosidase was carried out in duplicate using JetPei reagent (Polyplus, Illkirch, France). Forty-eight hours after transfection and miR-30a overexpression, luciferase and β-galactosidase activity was measured. The luciferase and beta-galactosidase activity were measured by luminescence in a microplate reader 5infinite M10000, Tecan, Mannedorf, Switzerland). Three independent biological replicates were performed.

### In vitro 3D human skin equivalents (SE) culture

SE cultures were prepared as described previously [44]. Briefly, fibroblasts from young donors were seeded at a final density of 250 000 cells/cm^2^ onto a dermal substrate (DS) made of chitosan-cross-linked collagen-glycosaminoglycan matrix [45]. This DS was grown in fibroblast medium supplemented with 50 mg/mL L-ascorbic acid (Sigma) and 10 ng/ml of EGF at 37°C in a 5% CO_2_ atmosphere, and the medium was changed every day for 21 days. For the preparation of SE, keratinocytes from young donor were seeded onto the DS on day 21. These submerged SEs were cultured for 7 days in keratinocyte medium and then raised at the air-liquid interface and cultured in a simplified kera-tinocyte medium containing DMEM supplemented with 10% FCS, 10 ng/mL EGF, 0.12 IU/mL insulin, 0.4 mg/mL hydrocortisone, and antibiotics. Samples were harvested after 35, 45, 55, 65, 75 and 100 days of total cell culture for histology, immunohistochemistry and genes expression studies as described [12]. For each cell culture condition and analysis, SEs were produced in triplicate.

### Reconstructed epidermis (RE) production

RE preparation was adapted from Le Provost et al., 2010 [13]. Briefly, 3.10^4^ fibroblasts were seeded on the outer face of the polycarbonate membrane of cell culture inserts (Millipore, Sigma-Aldrich, Saint-Louis, MO, USA). They were cultured for 2 days in DMEM/F12 supplemented with 10% fetal serum bovine (Gibco, Thermofischer, Waltham, MA, USA) and penicillin/streptomycin (Sigma, St-Louis, MO, USA). Then, 3.10^5^ keratinocytes from 3 different young donors and infected by pSLIK Venus control or pSLIK Venus miR-30a were seeded on the inner face of cell culture inserts and cultured for 3 days in DMEM/F12 supplemented with 5% fetal bovine serum (Fetal Clone II; Hyclone, Thermo Fisher Scientific, Waltham, USA), 0.2 ng/ml EGF (Gibco, Thermofischer, Waltham, MA, USA), 0.4 μg/ml hydrocortisone (Sigma, St-Louis, MO, USA), 5 μg/ml insulin (Sigma, St-Louis, USA), 8 ng/ml cholera toxin (Sigma, St-Louis, MO, USA), 2.10^−11^ M Tri-iodothyronine (Sigma, St-Louis, MO, USA), 24 μg/ml adenine (Sigma, St-Louis, MO, USA) and penicillin/streptomycin. To induce stratification and differentiation, keratinocytes were placed at the air/liquid interface and cultured for another 2 days in the same medium, except that EGF and adenine were omitted, the final calcium chloride (Sigma, St-Louis, USA) concentration was adjusted to 2mM, 50 μg/ml vitamin C and 0.1 μg/ml doxycycline were added. Cells were then cultured for another 12 days in the same medium, except that the fetal bovine serum concentration was reduced to 1%. During the immersion phase, the culture medium was changed every day. Three independent biological replicates were performed for each condition.

### Functional evaluation of barrier function

At the end of RE preparation, REs were held at room temperature without culture plate covers for 1 hour to remove any residual humidity. Then, trans-epidermal water loss (TEWL) was measured on Biox Aquaflux (Biox Systems Ltd, Bishop's Stortford, UK) according to the manufacturer's instructions. Three independent technical replicates were performed for each RE (3 REs per condition).

At the end of preparation, 200 μl of 1 mM Lucifer yellow (Sigma, St-Louis, MO, USA) was added on the RE surface. After incubation at 37°C for 6 hours, the Lucifer yellow concentration in the culture medium was measured by fluorescence in a microplate reader (Infinite M1000, Tecan, Männedorf, Switzerland) with excitation at 425 nm and emission at 550 nm. Three independent technical replicates were performed for each RE (3 REs per condition).

### Apoptosis TUNEL assay

A Click-iT® Plus TUNEL Assay (Thermo Fisher Scientific, Waltham, USA) was used to assess apoptotic cell rates, according to the manufacturer's instructions.

### Histology and immunofluorescence microscopy

RE samples were fixed in Zinc Formal-Fixx^TM^ (Thermo Fisher Scientific, Waltham, MA, USA) for 24 hours at 4°C, embedded in paraffin and then cut into 5 μm sections. After dewaxing and rehydration, tissue sections were either stained with HES to check morphology or permeabilized and blocked by 1% bovine albumin serum (BSA) + 10% goat serum + 0.3M glycine in PBS-T for 1 hour at room temperature. Sections were then incubated with primary antibody diluted in PBS + 0.1% goat serum overnight at 4°C: INVOLUCRIN (Sigma, St-Louis, MO, USA #I-9018, 1/200), K10 (Dako, Agilent, Santa Clara, CA, USA #M7002, 1/50), K1 (Covance, Princeton, NJ, USA #PRB-149P, 1/1,000), LORICRIN (Covance, Princeton, NJ, USA #PRB-145P, 1/1000), K14 (Novocastra, Leica, Nanterre, France #NCL-LL002, 1/200), AVEN (Sigma, St Louis, MO, USA #HPA020863, 1/100), IDH1 (Cell Signaling Technology, Danvers, USA #D2H1, 1/1,000). Secondary Alexa-488 and Alexa-546-conjugated anti-mouse or anti-rabbit (Molecular Probes, Eugene, Or, USA) was incubated 1 hour at room temperature. Nuclear counterstaining using DAPI was carried out. Sections were then mounted in Permafluor^TM^ Aqueous Mounting Medium (LabVision, Thermo Fisher Scientific, Waltham, MA, USA). Negative controls were performed by omitting the primary antibody.

To study LOX expression, tissue sections were etched and fixed in Bouin's fixative (Microm Microtech, Francheville, France) and then LOX expression was detected as previously described [46]. Ki-67 expression was detected as previously described [12]. Image acquisition was performed using a Nikon microscope (Nikon TE300, Champigny-sur-Marne, France) with a coolsnap fx CCD camera (Photometrics, Tucson, AZ, USA) with MetaVue software (Universal Imaging Corporation, West Chester, PA, USA).

### *In situ* hybridization of miR-30a

The assay was performed on young or aged skin and on RE paraffin sections as described previously [47]. On skin paraffin sections, hsa-miR-30a-3p, hsa-miR-30a-5p and scramble probes (Exiqon, Qiagen, Woburn, MA, USA) were used at 40 nM. On RE sections, these probes were used at 100nM. A LNA U6 positive control was used at 1 nM. The anti-peroxydase reaction was run for 24h at 30°C.

### Image analysis

Image analysis was performed using ImageJ software. The parameters of interested were Ki-67 nucleus positive cells and the surface area of K14, K1, K10, INVOLUCRIN, LORICRIN, LOX, IDH1 and AVEN. Quantifications were performed as previously described [12]. Data were normalized either by basement membrane length or by epidermal area.

### Statistical analysis

All data are represented as means for at least 3 independent experiments. Statistical significance was calculated by a two-tailed Student's t-test for unpaired samples to compare 2 groups and by one-way analysis of variance (ANOVA) to compare 3 or more groups. All statistical analyses were performed using Prism (version 6.0, GraphPad Software Inc., San Diego, CA, USA). All results are expressed as mean ± SD. Mean differences were considered to be significant when P<0.05. *P<0.05, **P<0.01, ***P<0.001, ***P<0.0001 (ns = non-significant).

## SUPPLEMENTARY MATERIALS FIGURES



## Abbreviations

UV: Ultra Violet
QPCR: Quantitative Polymerase Chain Reaction
SE: Skin Equivalent
RE: Reconstructed epidermis
HES: Hematoxylin Eosin Saffron
MRE: MicroRNA Responding Element
TEWL: Trans-Epidermal Water Loss
MOI: Multiplicity Of Infection
SD: Standard Deviation
HPK: Human Primary Keratinocytes
