# Supplementary material for: An expression screen for aged-dependent microRNAs identifies miR-30a as a key regulator of aging features in human epidermis
Source: Aging (Albany NY). 2017 Nov 19;9(11):2376–96. doi: 10.18632/aging.101326 (PMC5723692; doi:10.18632/aging.101326)
Supplement: Supplementary file 1 [file aging-09-2376-s001.pdf]

SUPPLEMENTARY MATERIAL

SUPPLEMENTARY FIGURES

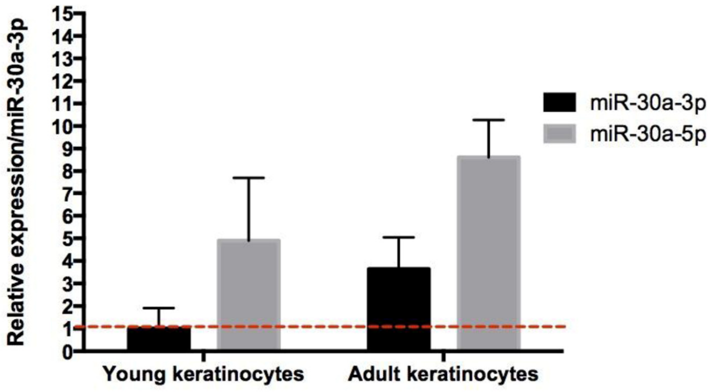

Figure S1. Relative expression of miR-30a-3p and miR-30a-5p in human keratinocytes. MiR-30a-3p and miR-30a-5p expression levels were measured by q-PCR on cultured keratinocytes from young and aged donors. Data were calculated using miR-30a-3p expression levels as a reference. Results are mean +/- SD from three independent samples.

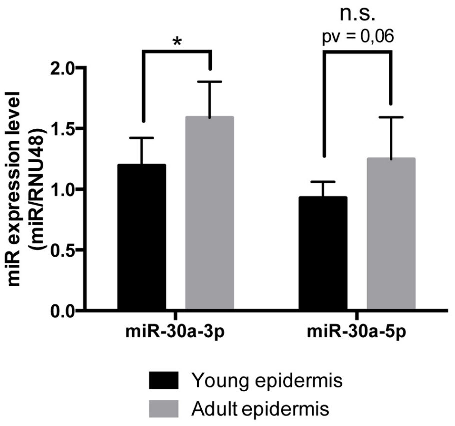

Figure S2. Relative expression of miR-30a-3p and miR-30a-5p in in young and aged epidermis. MiR-30a-3p and miR-30a-5p expression levels were measured by q-PCR on epidermis RNA samples from young and aged donors. Data were calculated using miR-30a-5p expression levels as a reference. Results are mean +/- SD from three independent samples. *P<0,05.

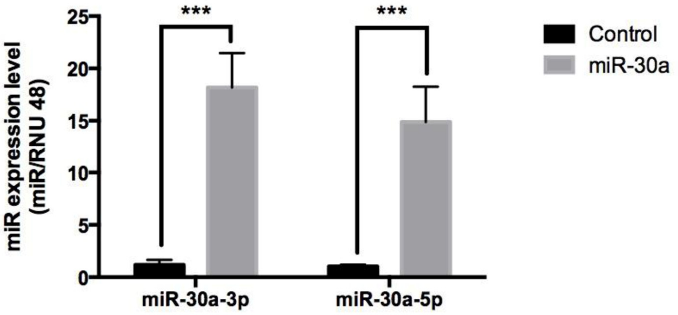

Figure S3. MiR-30a-3p and miR-30a-5p level in REs overexpressing miR-30a. Keratinocytes were infected with lentiviral vector particules of pSLIK-Venus control or pSLIK-Venus miR-30a, treated by doxycyclin and then used to generate reconstructed epidermis. MiR-30a-3p and miR-30a-5p expression levels were measured on total REs RNA sample by quantitative-PCR. Results are mean +/- SD from three independent samples. ***P<0,001.

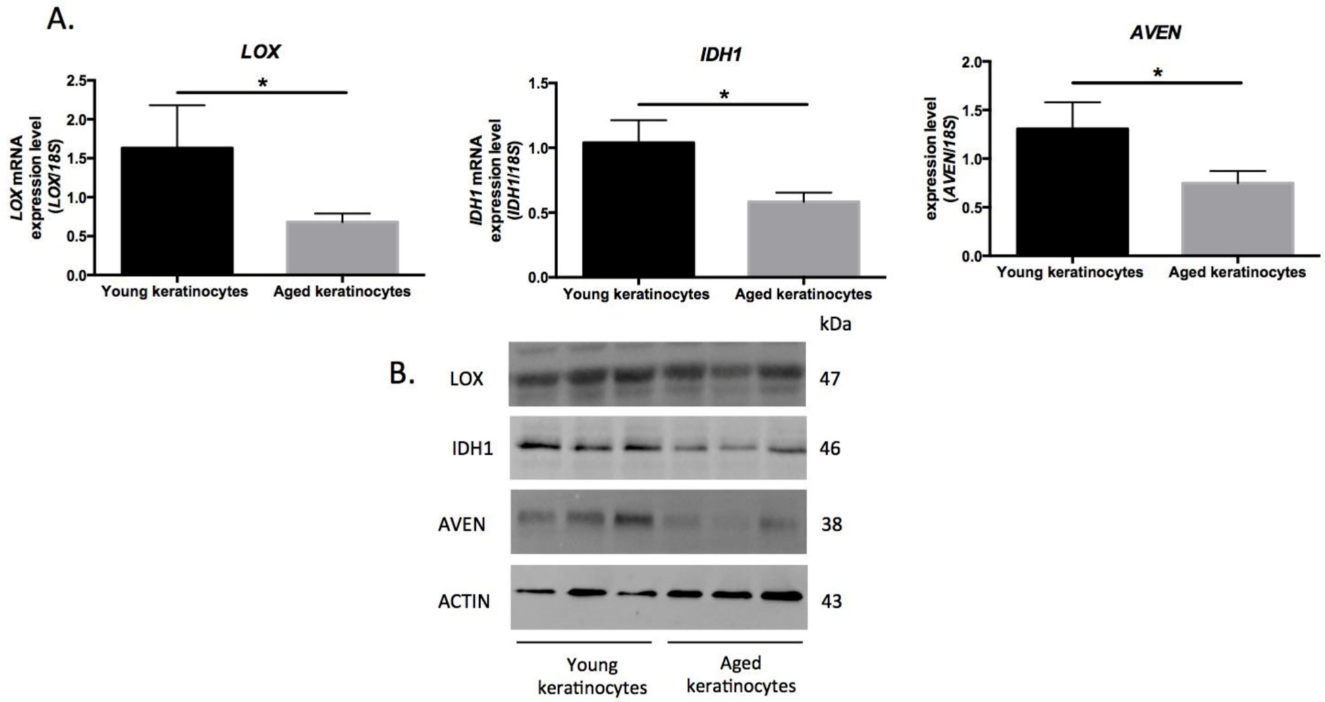

Figure S4. LOX, IDH1 and AVEN expression in keratinocytes from young and aged skin. (**A**) The expression level of AVEN, IDH1 and LOX transcripts were evaluated by qPCR in RNA samples from keratinocytes prepared from young or aged skin. Results are mean +/- SD from 8 independent samples. *P< 0,05. (**B**) The expression levels of AVEN, IDH1 and LOX proteins were evaluated by western blotting in cultured keratinocytes prepared from young or aged skin. Actin was used as a loading control.

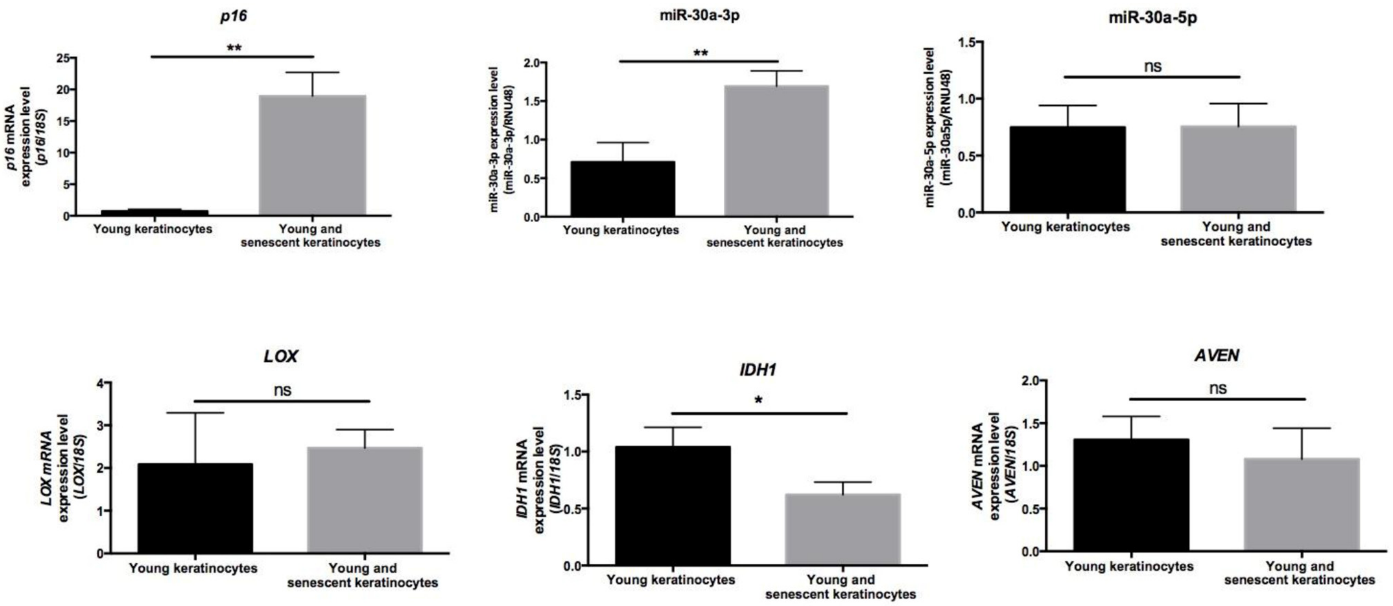

Figure S5. *P16*, miR-30a-3p, miR-30a-5p, LOX, IDH1 and AVEN expression in normal and replicative senescent keratinocytes from young skin The expression level of miR-30a-3p, miR-30a-5p, *AVEN*, *IDH1* and *LOX* transcripts were evaluated by qPCR in RNA samples from keratinocytes prepared from young skin at early passage (passage 2) and replicative senescent keratinocytes (passage 6). P16 was used as a marker of senescence. Results are mean +/- SD from three independent samples. *P< 0,05. **P< 0,01. ns : non significant.

SUPPLEMENTARY MATERIALS AND METHODS

List of primers used for QPCR

Gene	Gene ID	Primer	Primer Sequence
AVEN	57099	Reverse 5'	GCCAAGCTTGCATGCCTTGTTTGCATCTA-3’
		Forward5'	ATGAGCTCGCAGCTTGTTGCATTTGTTT-3’
IDH1	3417	Reverse 5'	GCCAAGCTTCATGTTCACAAAGGTGGCAAT -3’
		Forward5'	ATGAGCTCTCAAACTAGCTCAGGCCAAA-3’
LOX	4015	Reverse 5'	TTGGTCGGCTGGGTAAGAAAT-3’
		Forward5'	GGATACGGCACTGGCTACTTC-3’
18S	100008588	Reverse 5'	CGATGCGGCGGCGTTATT-3’
		Forward5'	CCTGGTGGTGCCCTTCCGT-3’

List of primers used for the amplification of the target genes 3’-UTR

Primers	Restriction enzyme	Sequences
3’ UTR AVEN forward	SacI	5’- ATgagctcGCAGCTTGTTGCATTTGTTT-3’
3’ UTR AVEN reverse	HindIII	5’-GCCaagcttGCATGCCTTGTTTGCATCTA-3’
3’ UTR IDH1 forward	SacI	5’-ATgagctcTCAAACTAGCTCAGGCCAAA-3’
3’ UTR IDH1 reverse	HindIII	5’-GCCaagcttCATGTTCACAAAGGTGGCAAT-3’
3’ UTR LOX forward	SacI	5’-ATgagctcATGGACACATCTGGTGCTGA-3’
3’ UTR LOX reverse	HindIII	5’-GCGaagcttCTGCCCATGGGAAAGATAAA-3’

List of primers used for the mutagenesis of the potential miR-30a MRE

Primers	Sequences
mutAVEN forward	5'-CAACATAGCCAGTGTCAGCATAGCAGATGC-3'
mut AVEN reverse	5'-GCATCTGCTATGCTGACACTGGCTATGTTG-3'
mut IDH1 f - site 1	5' GGTAACTAGGTCTACAGGTCATTTTTCTGTGTTACAC-3'
mut IDH1 r - site 1	5'-GTGTAACACAGAAAAATGACCTGTAGACCTAGTTACC-3'
mut IDH1 f- site 2	5'-GAGTTTATCTTTTCTATAAGTCAGCCTTTTTCTTATATATAC-3'
mut IDH1 r - site 2	5'-GTATATATAAGAAAAAGGCTGACTTATAGAAAAGATAAACTC-3'
mut LOX forward	5'-CCCTATATAAAAAGTATGTCATTTAAAAAATTAGTAG-3'
mut LOX reverse	5'-CTACTAATTTTTTAAATGACATACTTTTTATATAGGG-3'
